# Testing the Prey-Trap Hypothesis at Two Wildlife Conservancies in Kenya

**DOI:** 10.1371/journal.pone.0139537

**Published:** 2015-10-21

**Authors:** Marc Dupuis-Desormeaux, Zeke Davidson, Mary Mwololo, Edwin Kisio, Sam Taylor, Suzanne E. MacDonald

**Affiliations:** 1 Department of Biology, York University, Toronto, Ontario, Canada; 2 Marwell Wildlife, Winchester, United Kingdom; 3 Lewa Wildlife Conservancy, Isiolo, Kenya; 4 Borana Wildlife Conservancy, Kenya; 5 Department of Psychology, York University, Toronto, Ontario, Canada; U.S. Geological Survey, UNITED STATES

## Abstract

Protecting an endangered and highly poached species can conflict with providing an open and ecologically connected landscape for coexisting species. In Kenya, about half of the black rhino (*Diceros bicornis)* live in electrically fenced private conservancies. Purpose-built fence-gaps permit some landscape connectivity for elephant while restricting rhino from escaping. We monitored the usage patterns at these gaps by motion-triggered cameras and found high traffic volumes and predictable patterns of prey movement. The prey-trap hypothesis (PTH) proposes that predators exploit this predictable prey movement. We tested the PTH at two semi-porous reserves using two different methods: a spatial analysis and a temporal analysis. Using spatial analysis, we mapped the location of predation events with GPS and looked for concentration of kill sites near the gaps as well as conducting clustering and hot spot analysis to determine areas of statistically significant predation clustering. Using temporal analysis, we examined the time lapse between the passage of prey and predator and searched for evidence of active prey seeking and/or predator avoidance. We found no support for the PTH and conclude that the design of the fence-gaps is well suited to promoting connectivity in these types of conservancies.

## Introduction

In many parts of Africa, including in Kenya, there is an increased reliance on electrical fencing to protect wildlife and reduce human-wildlife conflicts [1–4], including in very large protected areas such as the Abedare Conservation Area [5, 6] and an ambitious project to enclose the Mount Kenya Forest Reserve with a 500 km electrified fence [7]. Lion (*Panthera leo*) and other predators can thrive within small fenced reserves [8]. Population numbers can be close to their estimated carrying capacity and prey abundance regulates space use and density [9, 10]. Although fencing is viewed as the most effective way to protect wildlife and reduce human-wildlife conflicts [11], fences come with a long list of drawbacks. Fencing wildlife causes mortality as animals can get entangled and killed while attempting to leave the fenced habitat [12–14]. Fencing also has many secondary drawbacks that can affect long-term population viability, including reduced access to resources [15–17], and the creation of edge effects [5, 18, 19]. Further, fencing is expensive to install and maintain as elephant often break their way through fencing [1, 4, 20] leading to costly repairs and potential human-wildlife conflict.

Wildlife managers attempt to mitigate these shortcomings by designing better fences [21], creating effective linkages between protected habitat [18, 22], reconnecting habitat by removing certain portions of fencing [23] and ensuring minimal encroachment from agriculture, urban development or roads [24–27]. Biologists emphasize creating connected landscape systems, on private and public lands, to ensure long-term persistence of highly mobile species [28–31]. Purpose-built fence-gaps permit some landscape connectivity for migration and dispersal. However, linkages and other connecting structures necessarily funnel animal movement into narrow areas that predators could learn to exploit due to the spatial predictability of prey passage. Due to this funnelling of movement, there is a concentration of spoor near the fence-gaps, which creates depositional odour trails that can be detected and followed by predators [32]. Predators do not necessarily have to kill at the fence-gaps but could use these cues to track prey further away from the crossing structures. For example, spotted hyena (*Crocuta crocuta*) use olfaction for hunting and will follow migrating prey for long distances [33] and can run down prey in an active chase for up to 4km [34].

The prey-trap hypothesis (PTH) has been advanced as a possible negative consequence of highway crossing structures by suggesting that predators can improve their predation success by hunting in and around these high prey traffic areas [35–37]. Although the empirical evidence is weak [38], anecdotal [39, 40] or unsupportive [35, 41, 42], there is evidence that fencing can lead to behavioural changes in some predators. For example, wild dog (*Lycaon pictus*) will incorporate fences into their hunting strategy to significantly increase their ability to take down large prey [43–45]. These studies raise fundamentally interesting questions that have yet to be fully tested in different ecosystems although the possibility of prey-traps developing at passageways has been raised [46]. Our research is the first to examine and formally test the PTH in a fenced conservancy equipped with fence-gaps to allow the passage of wildlife and is the first to test the PTH in an African savannah ecosystem.

The objective of this study was to test if predation events clustered near the fence-gaps and if we could detect active hunting or tracking at the fence-gaps. Successful management of migratory species within fenced conservancies depends on wildlife crossing structures acting as safe passageways in and out of suitable habitat, enhancing connectivity and long-term survival. Therefore, it is critical to verify that the connecting structures do not have any unforeseen negative consequences.

## Study Site

We tested the PTH at ten fence-gaps on the Lewa Wildlife Conservancy (25,000ha UTM 37 N 326024 25124, www.lewa.org), and at the adjacent Borana Conservancy (12,000ha, UTM 37 N 309280 24777, www.borana.co.ke) near Isiolo, Kenya. The properties comprise approximately 37,000 ha of electrically fenced wildlife refuge within a larger mixed habitat matrix that includes many small plot agricultural and pastoral communities, roads, towns, farms, and other conservancies. Both conservancies support the full breadth of the Eastern African savannah wildlife.

**Fig 1:**
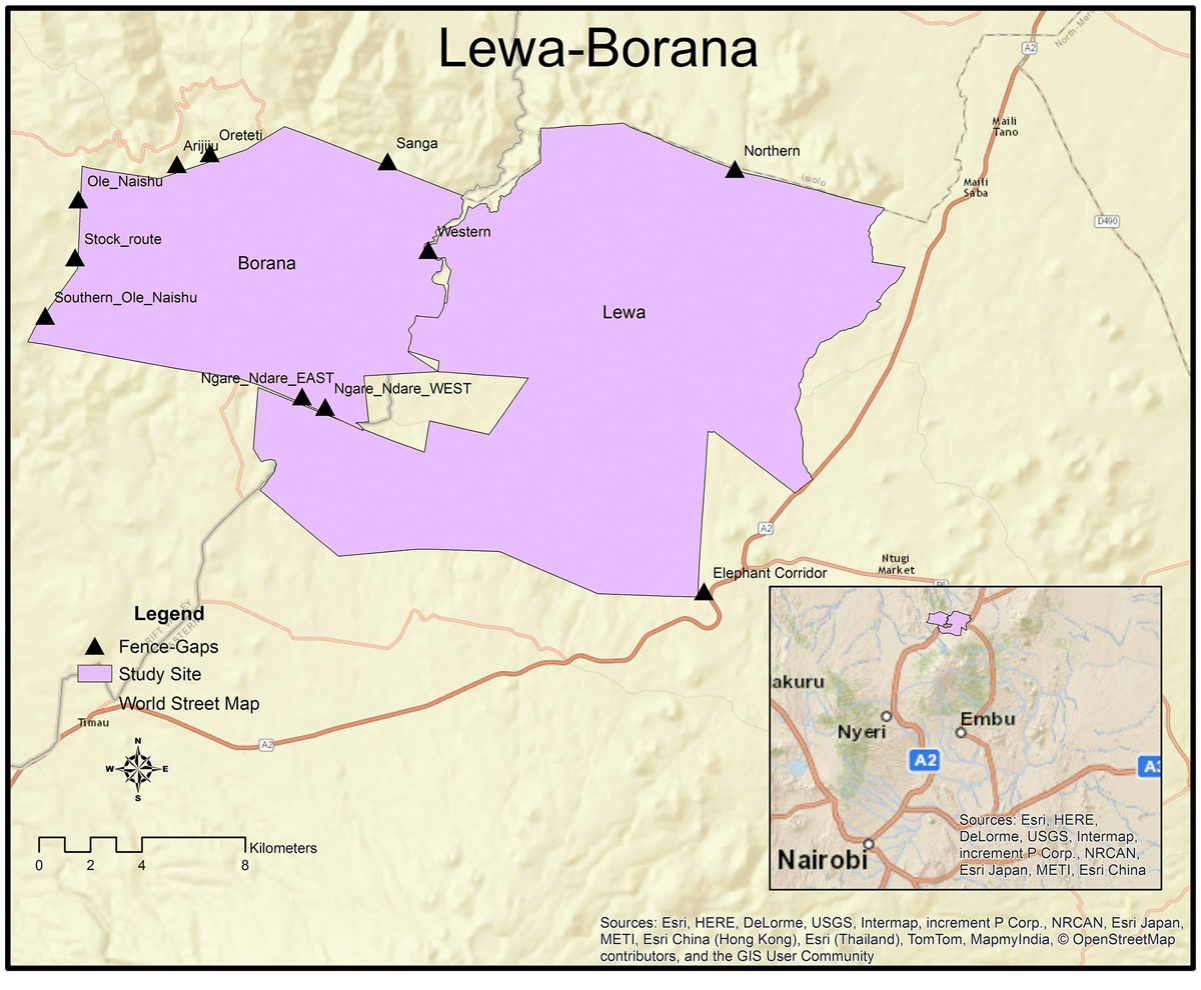
Lewa and Borana fence-gaps locations.

The vegetation of Lewa and Borana is classified as a mix of Northern Acacia-Commiphora bushlands and thicket [47] with significant areas of savannah. A 142 km long, two-meter high fence, consisting of twelve-strand of alternating electrified and grounded wires surrounds Lewa. The Northern fence-gaps measures approximately 30 m and leads to an unfenced pastoral area (Leparua community). The Western fence-gap measures approximately 20 m and joins the Borana Conservancy. The fence-gaps serve primarily to let elephant (and other migratory species) move through the fence and in and out of the conservancy. Lewa also has a fence-gap to the South that leads to a 14 km long elephant corridor linking Mt-Kenya and Lewa [26]. Borana has nine fence-gaps, including the shared fence-gap with Lewa (see Fig 1). Borana’s fence-gaps lead into different pastoral and agricultural community lands. Borana maintains 54 km of electric fence line that varies including 42 km of the same type of two-meter high twelve-strand variety, 4km of shorter elephant fencing (with electric “ticklers”- stinging wires protruding approximately one meter at a perpendicular angle to the fence- designed to discourage fence-breaking) and 8 km of double fencing, i.e. where both types of fences are used together. Borana’s fence-gaps measure between 5 m and 1000 m (Ngare Ndare gap) in width.

**Fig 2:**
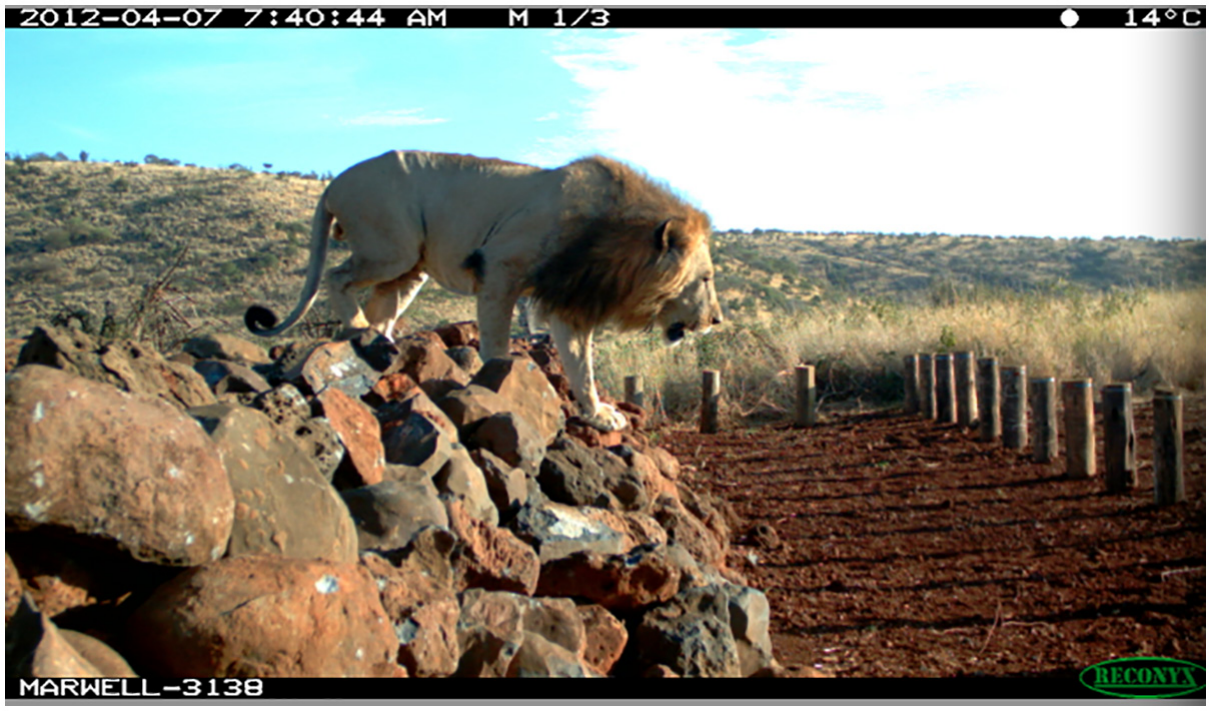
Photo of a lion crossing one of Lewa’s fence-gaps. The combination of the rock wall with the bollards is used to restrict rhino escaping from the conservancies into non-protected areas.

The design of the fence-gaps varies considerably between conservancies. Borana’s fence-gaps were designed primarily to permit easy access to community cattle, whereas Lewa’s fence-gaps were specifically designed to restrict rhino but permit other wildlife passage. As such, the Lewa design exploits a few of the unique anatomical features of the rhino, (i.e. short legs and wide body) by using a low wall constructed out of loose stones paired with a series of low bollards (see Fig 2). The placement of the bollards makes it very difficult for an adult rhino to squeeze through and the rock wall acts as a further obstacle should a smaller individual make it past the bollards. Species using the Lewa fence-gaps usually slow their pace and cross the fence-gap cautiously, but without much difficulty.

## Methodology

In order to test if predators learned to exploit the passage of prey at the fence-gaps, we captured baseline data measuring the volume of traffic through the fence-gaps using camera-traps. We then performed spatial analyses of predation events as well as a temporal analysis of predator and prey movements at the gaps.

### Baseline movement data

We used remotely triggered cameras (Reconyx RC60HO Hyperfire, Holmen, WI) to capture movement data at the fence-gaps in order to verify that there was regular prey traffic volume. Each fence-gap had at least one camera; all camera fields of view were perpendicular to the direction of wildlife travel. We mounted cameras in “elephant-proof” custom built steel housings. Cameras were configured for a three exposure burst upon being triggered by their inbuilt motion detectors and set for rapid-fire to ensure continuous shooting for as long as their sensors detected motion. Images were stored on 32GB Secure Digital (SD) memory cards. From time to time photographs were uploaded into a central database for later analysis.

### Spatial Analysis

We collected predation data from Lewa dating back to 2004 (702 kills) and from Borana since 2010 (115 kills). The collection of carcass data is a strategic imperative for all 283 field staff at the study site and, as of 2015 included anti-poaching patrollers, rhino rangers, safari guides, fencers, trackers, herders, and research personnel. Anti-poaching patrols (n = 62, as of 2015) are armed and can travel by foot or by vehicle. These patrollers are deployed 24-hours a day, seven days a week and patrol mostly near the access points, roads, fence-line and fence-gaps but can access all of the study site, including by vehicle, plane and helicopter. They also patrol the entire fence-line searching for security breaches and elephant damage to the electrical fence on a regular basis. On Lewa, rhino rangers (n = 81) track on foot each individual black rhino, 24-hours a day and report position on a daily basis via radio. These rangers follow the rhino everywhere they go on the conservancy. Safari guides (n = 32) work mostly out of vehicles, but also on foot and sometimes on horseback and report kills to the main office. Further, it is important to note that the safari guides are highly incentivized to locate fresh kills for the tourists. There are also fencers (n = 38) that patrol the 150km perimeter and 98 km exclusion fences for damage. Trackers (n = 2) read the spoor of mammals that have used the fence-gaps on a daily basis and would report any carcasses found near the fence-gaps. As the study site supports local cattle herds at various times of the year, herders (n>55) also come across carcasses when tending their cattle. Finally, researchers (n = 13) at the study site are actively monitoring rhino, elephant, ungulates and predators by road vehicle or by plane. It is also noteworthy that the study site has an extensive road network (in excess of 800 km of internal tracks and roads) and that no point is more than 1 km away from a track or road. The study area is thus well monitored and mortality data are considered representative.

We established the exact location of the predation events by assigning a set of GPS coordinates to the descriptive physical locations of each reported carcasses. We used only verified predator kills in our data set. We assigned a cause of death to every carcass found and animals that had died of other causes (unverified as predator kills, drought, electrocution, etc.) were not included in our analysis.

Starting in April 2014, Lewa began using a cluster point method (nearest neighbour) as described by Davidson et al. [48] as an additional search method focused on certain lion prides. Researchers collared five lion groups with GPS radio collars and monitored potential kill sites by identifying locations where the lion activity clustered in excess of four hours.

The detection of carcasses in the field is sensitive to prey size, as larger carcasses are easier to detect and last longer increasing the probability of discovery, therefore smaller prey is likely under-represented in our sample [48].

#### Proximity Analysis

Using the Pearson’s chi-squared test statistic, we tested the hypothesis that the level of kills at or near the fence-gaps was not significantly different than what could be expected elsewhere on the conservancies by comparing the actual density of kills near the gaps with the expected density of kills in the rest of the conservancy.

We created concentric ring buffers around the fence-gaps at various radii (500, 1000 and 2000 m) and intersected those buffers with the shape of the conservancy to calculate the captured areas. We then measured the number of kills recorded in each buffer and compared that number to the expected number of kills on each conservancy for an area of equal size. The total buffer area at each radius distance represents the sum of the areas captured within the defined radius at all the gaps on each respective conservancy.

Stander [49] reported that the majority of lion kills (73%) were recorded after short chase distances (less than 20m) and the rest (27%) were between 20m and 150m. Scheel [50] observed hunting distances of up to 200m. Although Holekamp et al. [34] reported that hyena chased down their prey over distances ranging from 75 m up to 4 km. For our analysis, given that ambush predators dominate our predation data set, (lions, cheetah and leopards representing approximately 81%, 8% and 6%, resp. of the kills in our data) we report the search radius analysis up to 2000m.

#### Clustering

We tested for clustering of the predation locations at the conservancy level by calculating separate global measures of clustering, the Getis-Ord General G statistic, and Global Moran’s I. For the clustering analyses, we aggregated individual incident data points by selecting locations that were within a 100m radius of each other, to match with the precision tolerances of the data collection methods, approximately +/- 50m of each data point.

The Getis-Ord General G measures the degree of clustering of the locations with high/low predation counts over the whole landscape. The General G returns a global z-score, if it is significantly positive then the areas of high predation tend to cluster with other areas of high predation. If the z-score is significantly negative then areas of low predation are clustered with other areas of low predation. The null hypothesis is complete spatial randomness.

Global Moran’s I measures spatial autocorrelation based on the location and the weight of each data point (weight based on the number of reported kills for that location) and compares it against randomness. If the z-score is significantly positive then the spatial distribution is more spatially clustered than would be expected under a random spatial process. If the z-score is significantly negative, then the spatial distribution is more dispersed than would be expected under a random scenario. We calculated the spatial autocorrelation for a number of incremental neighborhood sizes. We selected the smallest distance needed to ensure that all weighted predation locations had at least one neighbor as our starting point. We created a table of z-scores from which we selected the neighborhood distance that corresponded to the first peak in significant z-scores (i.e. where the next incrementally larger neighborhood had a smaller z-score). The selected neighborhood size represents the smallest neighborhood distance where significant spatial autocorrelation is occurring, i.e. the smallest distance where the spatial processes promoting clustering are most pronounced.

#### Hot Spot Analysis (Getis-Ord Gi*)

In addition to calculating a global clustering measure as above, we also tested if any of the individual predation locations were significantly different from the others by performing a hot spot analysis using a local statistic, the Getis-Ord Gi*.

The Gi* statistic returns a z-score for each location in the data set. Significantly positive scores indicate statistically significant clustering of locations with high predation counts (i.e. the hot spots) and significantly negative scores indicate statistically significant clustering of locations with low predation counts (i.e. cold spots). We performed the hot spot analysis (Getis-Ord Gi*, zone of indifference conceptualization) on the aggregated incident data (used in the incremental spatial autocorrelation) and identified the statistically significant hot and cold spots. We used the first peak in significant autocorrelation values, as computed with Global Moran’s I above, as the distance input factor in the Hot Spot Analysis, since the first peak is at the spatial scale representing the smallest distance with the most pronounced clustering.

All spatial analysis was done in ArcMap 10.1, (ESRI, USA).

### Temporal Analysis

For the temporal analysis we used a different data set based on the time stamp from each photo. The camera-trap data was collected at the busiest fence-gap, at Lewa’s Northern gap, between January 1, 2010 and December 31, 2011. We defined a “species crossing event” as the photographic record of the passage of one or more individuals of a species going in the same direction (inbound or outbound) through the fence-gap. For every crossing event, we recorded date, time, species detected, direction of travel and number of individuals of that species crossing in the same direction. We selected a subset of these data where the crossing of a prey species was followed by the crossing in the same direction of a predator species. We calculated the time differential of these paired crossing events and designated this time differential as the HUNT variable. We also paired the crossing of the predator to the next prey crossing. We calculated the time differential between these paired crossings and designated this time differential as the AVOID variable. We tested for normality and performed a paired difference test to compare the means of HUNT and AVOID. To test whether prey passage modified predator behavior, we followed Ford and Clevenger [35], where we compared the mean time lapse between the passage of prey followed by predator (HUNT) versus the inverse, the passage of predator followed by prey (AVOID). We expected that if predators were actively using the gaps to pick-up scent tracks and if prey species were actively avoiding using the gaps after predator passage the HUNT time interval should be shorter than the AVOID time interval. We tested the HUNT and AVOID variables for normality and performed a paired differences test on the means.

## Results

### Baseline Camera-trapping data

**Fig 3:**
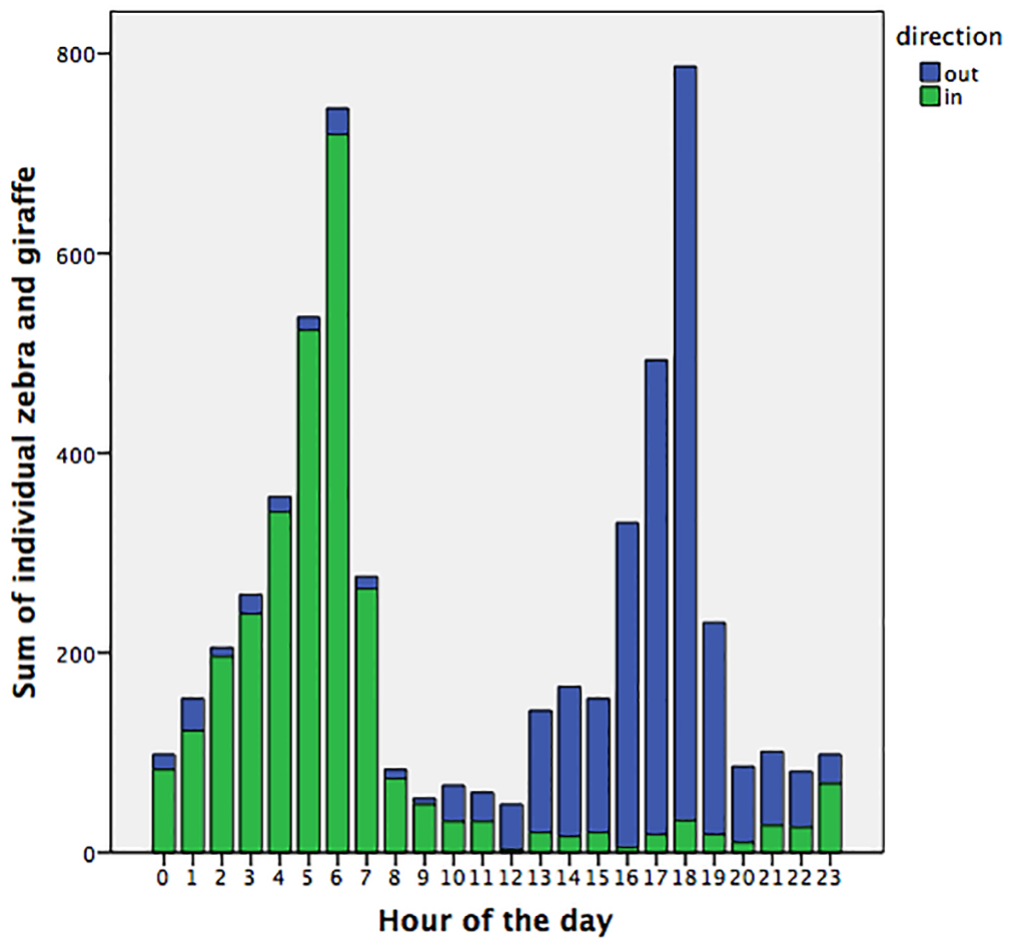
Predictability of prey passage through Lewa’s Northern fence-gap (2010–2011). *Equus quagga* (n = 2773, into Lewa = 1488, out of Lewa = 1285), *Equus grevyi* (n = 422, into Lewa = 245, out of Lewa = 177), *Giraffa camelopardalis reticulata* (n = 2413, into Lewa = 1201, out of Lewa = 1212)

We monitored wildlife movements through the various fence-gaps using camera-traps. During 2010–13, we recorded in excess of 50,000 mammals of 34 species crossing through the Lewa fence-gaps (46,065 crossings at the Northern fence-gap, 1,176 at the Southern fence-gap, 3,427 at the Western fence-gap joining Lewa and Borana). The most frequent prey species using the fence-gaps were elephant (n = 20,335), plains zebra (*Equus quagga*) (n = 17,292), reticulated giraffe (*Giraffa camelopardalis reticulata*) (n = 7,879), and Grevy’s zebra (*Equus grevyi*) (n = 1,930). The most common predators using the fence-gaps were spotted hyena (n = 2,055), lion (n = 167), and leopard (*Panthera pardus*) (n = 68). Temporal analysis of the movement data showed strong predictability of prey through the busiest fence-gaps (see Fig 3). We also conducted a brief survey of Borana’s other fence-gaps and found in excess of 800 mammal passages in a few months of camera-trapping.

### Spatial Analysis

#### Proximity Analysis

We recorded two kills within 500m of the crossing gaps, nine kills within 1000m and 46 kills within 2000m over both conservancies. We calculated the chi-squared statistic for the probability of the kills being over or under represented in the sampled area near the gaps. As shown in Table 1, predation events were significantly under-represented near the gaps.

**Table 1:** Proximity Analysis (2004–2014) of the predation events by conservancy.

	Total Kills	Kills in buffer area	Buffer radius (m)	Total buffer area (km^2^)	Expected kills in buffer area	Pearson’s Chi-squared	p-value (df 1) <
Lewa	702	15	2000	19.40	53	26.87	0.0001 *
		2	1000	5.07	14	10.43	0.001*
		1	500	0.98	3	1.11	0.292
Borana	115	22	2000	43.10	33	3.89	0.048*
		5	1000	12.75	12	3.90	0.048*
		1	500	3.11	3	1.31	0.252

*Pearson’s chi-squared values where the recorded kills in the buffer zones around the fence-gaps differed significantly from the expected values (p<0.05).

#### Clustering

We tested the data at each conservancy for High/Low Clustering by calculating the Getis-Ord General G statistic. We found that predation locations were significantly clustered on both conservancies (see Table 2).

**Table 2:** Overall Clustering Analysis-using collected predation events at 100m tolerances.

	Observed mean distance between kill locations (m)	Maximum distance between kill locations (m)	Getis-Ord General G statistic	z-score	p-value>
Lewa	495	2052.2	0.07	3.10	0.002
Borana	614	2472.2	0.21	3.08	0.002

We performed an incremental spatial autocorrelation (ISA) analysis using Global Moran’s I statistic (zone of indifference conceptualization). We aggregated the data points that fell within the precision tolerance of 100m resulting in the 702 individual predation locations to aggregate into 304 weighted data points (weighted by incident count ranging from 1–15 individual predation events) on Lewa. Similarly, the 115 individual kill locations on Borana aggregated into a set of 89 weighted data points (ranging from 1–4 individual predation events).

We found that the first distance at which significant spatial autocorrelation occurred was at 3538 m on Lewa (z-score = 3.035, p<0.01) and at 4315 m on Borana (z-score = 2.576, p<0.01).

#### Hot Spot Analysis

**Fig 4:**
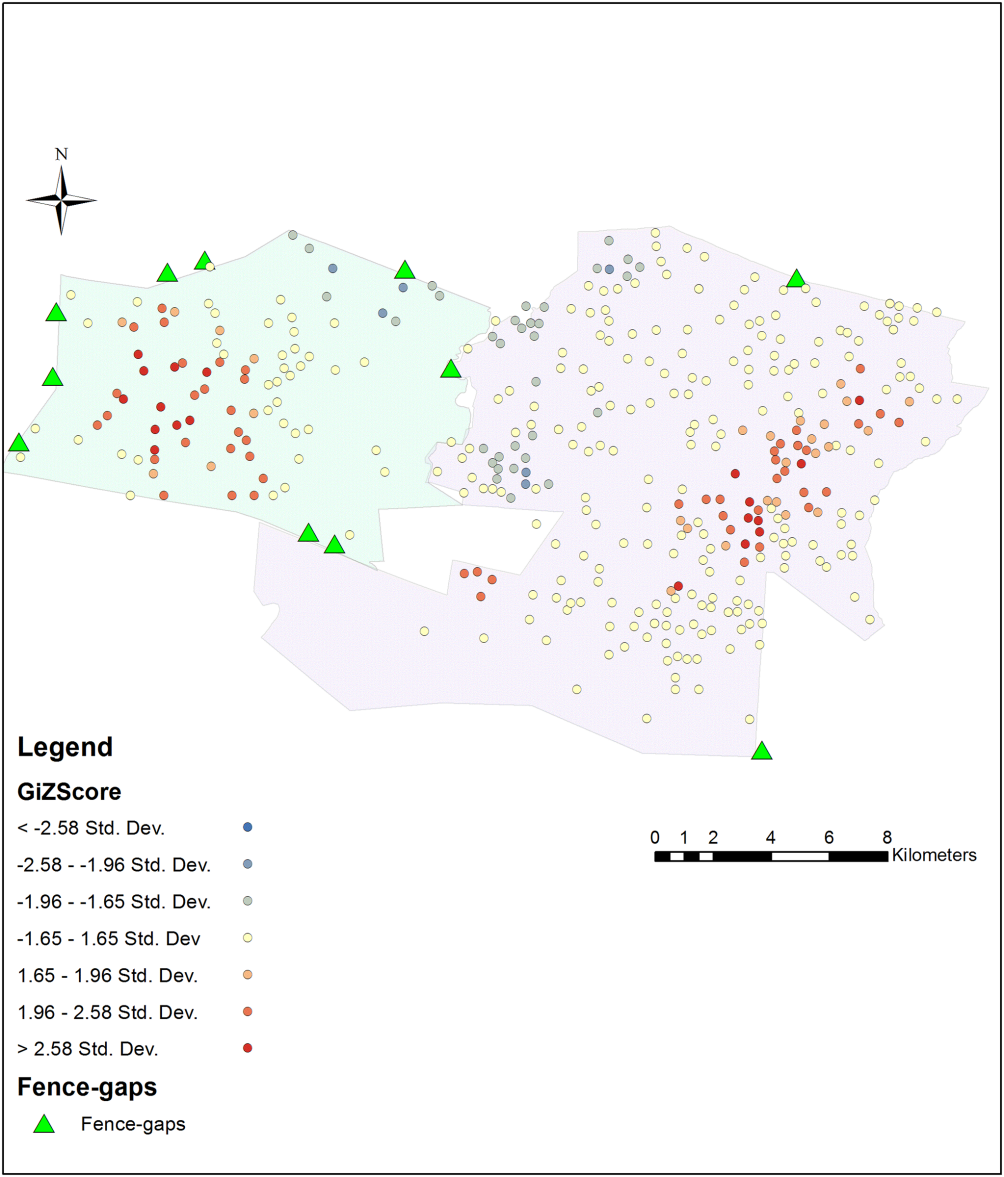
Hot Spot Analysis. Lewa and Borana calculated separately but shown on the same map. Predation events (2004–2014). Each predation location (collected to 100m tolerance) has its Getis-Ord Gi* statistical Z-score reported as a color ranging from blue (cold) to red (hot).

We calculated the Getis-Ord Gi* statistic for each point of aggregated data and mapped these points in Fig 4. Hot spots are represented in red, cold spots in blue. No hot spots were detected near the gaps.

### Temporal Analysis

For the temporal analysis we used a different data set based on the time stamp from each photo. The camera-trap data was collected at the busiest fence-gap, at Lewa’s Northern gap, between January 1, 2010 and December 31, 2011. During that time interval, we captured 4546 crossing events (14,194 individuals) including 444 crossing events that involved a predator species. Out of those 444, we recorded 217 HUNT and AVOID events. The range of HUNT was from 1 minute to almost 24 hours (mean = 3.77 hrs, median = 1.8 hrs, STD = 5.23 hrs, n = 217). The range of AVOID was from 1 minute to in excess of 22 hours (mean = 3.29 hrs, median = 1.3 hrs, STD = 4.54 hours, n = 217).

We tested both HUNT and AVOID for normality by performing a Shapiro-Wilk (SW) test and found that both variables were not normally distributed (HUNT: SW = 0.680, p<0.001, AVOID: SW = 0.689, p<0.001). We compared the means of HUNT and AVOID by performing a non-parametric paired difference test and found that the means of both variables were equal (Wilcoxon signed ranked test z = -0.652, p<0.514).

## Discussion

Using spatial analysis, we found no support for the prey-trap hypothesis as it related to fence-gaps at our study site. On the contrary, our data show significant under-representation of predation locations near the gaps. We found that predation events did cluster, but not near the fence-gaps. Cluster analysis found that the kill locations were focused near denser vegetation and watering holes [51]. We also identified many hot spots of predation activity, but none were near any of the fence-gaps.

Temporal analysis found no discernible difference between time intervals between the passage of predator followed by prey or prey followed by predator, i.e. showed no active hunting by predators or avoidance of predators by prey at the busiest fence-gap. However, this finding does not rule out that predators pick up scent trails just outside of camera range and start tracking the prey.

Based on general behavioural theory for large carnivores, we suggest that the lack of predation at the fence-gaps may be due to the risk of encountering humans [52], the condition of the prey using the fence-gaps and the potential hyper-vigilance of the prey due to the open nature of habitat at the gaps. Lion movement near the borders of protected areas is heavily influenced by the risk of encounters with humans. Lion will tend to avoid areas of high human traffic and times of high human activity [53, 54]. It is possible that the threat of encountering humans discourages hunting at the fence-gap locations. Human activity near the fence-gaps includes daily anti-poaching patrols, conservancy vehicle traffic, daily track observers, bi-weekly researchers checking camera traps, and occasionally tourists and curious community members. Poachers have also been detected passing through the fence-gaps. In addition, nomadic pastoral people frequently occupy homestead sites within 2 km of the fence-gaps. Their herding, wood and water gathering activities provide a constant flow of human and livestock traffic near these conservancy boundaries.

Lion and other predators suffer constant persecution outside of protected areas [55–58], in retaliation for livestock depredation, especially in areas of subsistence pastoralism [59]. Such evidence suggests that lion with home ranges spanning both protected areas and communal lands will not follow migrating wildlife herds out of the protected areas, but prefer to hunt inside the borders of the protected area. This combined with the regular presence of nomadic pastoralists adjacent to both conservancies’ boundaries may partly explain why predators avoid hunting near the fence-gaps and hunt in more central locations on the conservancies.

Lion discriminate for substandard (older, sick, infirm) individuals when selecting prey [60]. Substandard prey might not be able or willing to travel off the conservancies and thus might not encounter the fence-gaps. Given that our study sites have permanent water and forage resources, weaker animals do not have to travel outside the conservancies in order to survive. Work by Pratt [51] at our study site supports the findings of Valeix et al. [61] and Davidson et al. [62] respectively, that lion intensify their search for prey within 2km of watering holes and use these areas where prey congregate as hunting grounds and focus their searches in bushed grasslands and near watering holes, but that their kill success is highest in dense vegetation.

We also suspect that frequent traffic of elephant and giraffe might be a deterrent to hunting. Although lion will occasionally kill an elephant or a giraffe, they prefer prey species between 32 and 632 kg [63]. Prey in their preferred weight range abounds on both Lewa and Borana, making the gap sites less desirable as they attract constant traffic of mega herbivore species. Landscape features also affect where lion hunt, as lion prefer to hunt where prey is easier to catch versus areas where prey is more abundant [64]. Elephant feeding behaviour significantly modifies the landscape by transforming woody vegetation into grasslands [65, 66] and this phenomenon is extensive at our study site [67]. Areas where herds of elephant regularly pass through are often devoid of large trees and offer better visibility [68]. Many herbivores tend to use these more open habitats to reduce the risk of ambush [69]. Prey species have developed behaviours that protect against predation risks [70, 71] including hyper vigilance in the presence of lion [72, 73]. The vegetation at our study site varies from forest, to thick bushland, to grassland. The vegetation cover at the various fence-gaps varies according to their location, but open grasslands dominate at the two most highly used fence-gaps, the Northern and Western gaps. We suspect that the open nature of the immediate area near these fence-gaps might deter lion predation.

Thus, in conclusion, our findings do not support the argument for prey traps developing at fence-gaps. Our study site provides useful dynamics of robust predator and prey populations, and strong prey trap potential. It appears, that despite prey availability, predatory behaviour is not focused at these fence-gaps. Habitat structure, landscape topography, water availability elsewhere, density of vegetation, prey condition, and human activity appear to make the costs outweigh the benefits of expending predation effort at these locations at this time. However, management should remain vigilant for the development of prey-traps as predation dynamics may change over time. Given the trend towards fencing for conservation throughout Africa and the need to establish safe connecting routes between protected areas, we conclude that fence-gaps are a feasible wildlife management tool.
